# Efficacy and safety of neoadjuvant chemotherapy (NACT) with paclitaxel plus carboplatin and oral metronomic chemotherapy (OMCT) in patients with technically unresectable oral squamous cell carcinoma (OSCC)

**DOI:** 10.3332/ecancer.2021.1325

**Published:** 2021-12-02

**Authors:** Lakhan Kashyap1, Vijay Patil, Vanita Noronha, Amit Joshi, Nandini Menon, Kunal Jobanputra, Saswata Saha, Pankaj Chaturvedi, Shripad D Banavali, Kumar Prabhash

**Affiliations:** 1 Department of Medical Oncology, Tata Memorial Hospital, Mumbai 400012, India; 2 Department of Head and Neck Oncosurgery, Tata Memorial Hospital, Mumbai 400012, India

**Keywords:** neoadjuvant therapy, metronomic chemotherapy, paclitaxel-carboplatin, squamous cell carcinoma, oral cavity

## Abstract

A combination of maximum tolerated dose and metronomic chemotherapy schedule may lead to synergistic effects with acceptable toxicity. We assessed the efficacy and safety of this combination as neoadjuvant chemotherapy (NACT) in 14 patients with technically unresectable oral squamous cell carcinoma. They received NACT with paclitaxel-carboplatin and triple oral metronomic chemotherapy (OMCT) (methotrexate, celecoxib and erlotinib). Patients were assessed clinically and radiologically after a minimum of two cycles for resectability. Primary tumour site was buccal mucosa and oral tongue in 12 (86%) and 2 (14%) patients, respectively. The median number of NACT administered was three. The tumours of nine (65%) patients showed partial response and none of the patients had tumour progression. The tumours of nine patients (65%) were deemed resectable after NACT. Median progression free survival was 11.4 months (95% CI = 7.9–15 months) and median overall survival (OS) was not reached. OS at 15 months was 63.5% (95% CI = 37.8%–89.2%). Grade 3 or 4 haematological toxicities were seen in eight (57%) patients. Paclitaxel-carboplatin combined with OMCT is a well-tolerated and less resource intensive NACT regimen which leads to favourable resection rate and survival.

## Background

A chemotherapy regimen used in the neoadjuvant setting has been scheduled on the maximum tolerated dose (MTD) [1, 2]. MTD as determined in phase I/II study induces maximum tumour response with acceptable adverse effects. However, various tumour-related factors lead to the development of resistance to the MTD schedule. Chemo-resistance of the MTD schedule is mainly due to intrinsic tumour microenvironment and cancer stem cells. Furthermore, these factors often cause incurability of the cancer and recurrence of the tumour [3]. The MTD regimen may cause mobilisation of bone marrow derived cells and subsequent infiltration into tumour leading to accelerated repopulation [4]. Contrary to the conventional MTD approach, metronomic chemotherapy (MCT) entails continuous administration of cytotoxic drugs at low doses that cause minimal toxicities. MCT acts mainly through the inhibition of angiogenesis; however, other mechanisms are also involved including immune surveillance [5]. Combination of MTD and MCT schedule would lead to initial debulking of the tumour and subsequent inhibition of angiogenesis, this may lead to synergistic effects [6, 7]. This multitargeted chemo-switch schedule has been shown by Vives *et al* [8] to have an inhibitory effect on angiogenesis, cancer stem cells and tumour dissemination. This approach may also overcome the drug resistance of the MTD schedule.

Based on objective clinico-radiological factors, Patil *et al* [9] have defined a subgroup of borderline/technically unresectable oral squamous cell carcinoma (OSCC) where neoadjuvant chemotherapy (NACT) may lead to tumour downstaging and resection. The combination of a MTD and MCT schedule may be an attractive option for NACT in borderline/technically unresectable OSCC, especially when the administration of TPF (Docetaxel, Cisplatin and 5FU) regimen remains a challenge. We performed this study to assess the safety and efficacy of this combination approach. This regimen was offered as an alternative to TPF when it was not feasible either due to the logistics or comorbidities. Triple metronomic chemotherapy (methotrexate, celecoxib and erlotinib) was selected as the MCT schedule based on favourable response rate in platinum-refractory/early failure oral cancers [10]. Paclitaxel and carboplatin were selected as MTD schedules based on the efficacy and favourable safety profile of this combination [11, 12].

## Materials and methods

### Methodology

The Head and Neck Medical Oncology unit maintains a prospective NACT database for all patients undergoing NACT. This database was accessed for identifying these patients. All of these patients had borderline resectable/technically unresectable OSCC and were discussed in a multidisciplinary clinic. These patients were included based on following clinico-radiological factors: 1) peritumoral oedema above zygoma or involvement of high infratemporal fossa in patients with buccal mucosa as primary tumour site; 2) posterior extent of tumour till vallecula in patients with oral tongue as primary tumour site and; 3) up to 180⁰ nodal encasement of the internal carotid artery (ICA) in remaining patients [9]. Patients having tumour with nodal encasement of ICA more than 180⁰ or base of skull invasion were excluded. Patients were planned for NACT and assessment of response at 5–6 weeks after it. These patients had logistics issues in undergoing TPF/TP (Docetaxel and Cisplatin) regimen due to limited finances or bed availability, and hence this schedule was selected. All patients provided written informed consent prior to the administration of chemotherapy. The approval from institutional ethics committee was obtained for carrying out this retrospective study (TMC IEC approval number 900835).

Fourteen patients of technically unresectable OSCC were included. These patients received NACT with paclitaxel (175 mg/m^2^) plus carboplatin (area under curve (AUC) 5) every 3 weeks and triple oral metronomic chemotherapy (methotrexate 9 mg/m^2^ once a week, celecoxib 200 mg twice daily and erlotinib 150 mg once daily). Adverse events during NACT were graded and reported as per CTACE 5.0 (common terminology criteria for adverse events). Growth factor was used for secondary prophylaxis if the patient developed grade 3 neutropenia or febrile neutropenia in the preceding chemotherapy cycle. Dose reduction was done based on tolerance and toxicity in the previous cycle. Contrast-enhanced CT of head and neck was performed after two cycles of NACT and disease response was assessed using RECIST 1.1 (response evaluation criteria in solid tumours 1.1). Partial response was defined ≥ 30% decrease in sum of target lesions as compared to baseline while stable disease included <20% increase in sum of target lesions where partial response was not achieved. Subjective benefit based on patient’s self-assessment disease-related symptoms was also documented. After NACT further management of these patients was discussed in the multidisciplinary tumour board. If the tumour was resectable, then patients underwent surgery and received adjuvant chemoradiation up to 60 to 64 Gy with concurrent cisplatin 40 mg/m^2^ weekly or carboplatin AUC 1.5 weekly (in cisplatin unfit patients). If the tumour showed a partial response or stable disease to two cycles of NACT, however, it was still deemed technically unresectable, another one to two cycles of chemotherapy was administered provided the patient did not experience any grade 3 or higher toxicities (as per CTCAE 5.0) in previous cycles. If the tumour was considered unresectable even after the completion of three to four cycles of NACT, the patient could be treated with radical chemoradiation up to 70 Gy dose with concurrent cisplatin 40 mg/m^2^ weekly or carboplatin AUC 1.5 weekly (in cisplatin unfit patients). The decision regarding radical chemoradiation was based upon disease volume, performance status and tolerance to NACT. The patient who had the technically unresectable disease after NACT and was not deemed fit for radical chemoradiation received palliative chemotherapy.

### Statistical analysis

The data of all the patients were analysed for disease response, resectability, progression free survival (PFS), overall survival (OS) and adverse events. Disease response included either complete response (CR) or partial response (PR) as per RECIST 1.1. PFS was defined from the date of diagnosis to disease progression or death. OS was defined from the date of diagnosis to death. Patients who did not come for follow up in last 6 months and could not be contacted telephonically were considered as lost to follow up which was taken as an event for PFS and OS analysis. The response rate and resectability rate was reported as simple percentages with 95% confidence interval (CI). The Kaplan–Meir method was used for survival analysis. Follow up was estimated by reverse Kaplan–Meir method. SPSS 24 (IBM Corp. Released 2016. IBM SPSS Statistics for Windows, Version 24.0. Armonk, NY: IBM Corp.) was used for statistical analysis.

## Results

### Baseline characteristics

The baseline characteristic of patients is summarized in Table 1. The median age of patients was 38 years (range = 29 to 59 years) and 12 patients (85%) were male. Clinco-radiological factors used to identify borderline resectable or technically unresctable status of the tumour in these patients were as follow: 1) peritumoral oedema above zygoma (*n* = 5) or involvement of high infratemporal fossa (*n* = 5) in 72% patients with buccal mucosa as primary tumour site; 2) posterior extent of tumour till vallecula (*n* = 2) in 14% patients with oral tongue as primary tumour site; and 3) up to 180⁰ nodal encasement of the internal carotid artery (*n* = 2) in remaining 14% patients (Supplementary Table 1).

### Response

**Figure 1.**
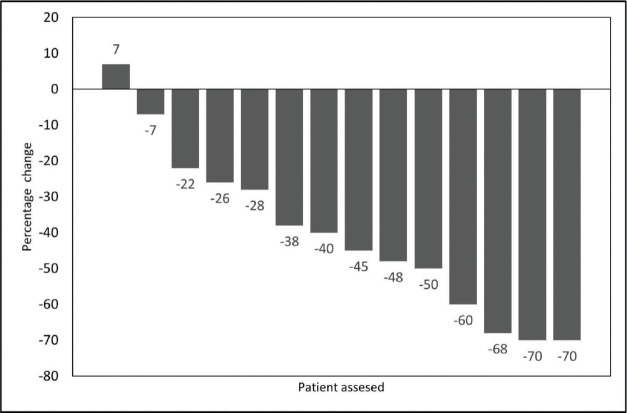
Waterfall plot showing depth of response as percentage change in measurable tumour of 14 patients.

**Figure 2.**
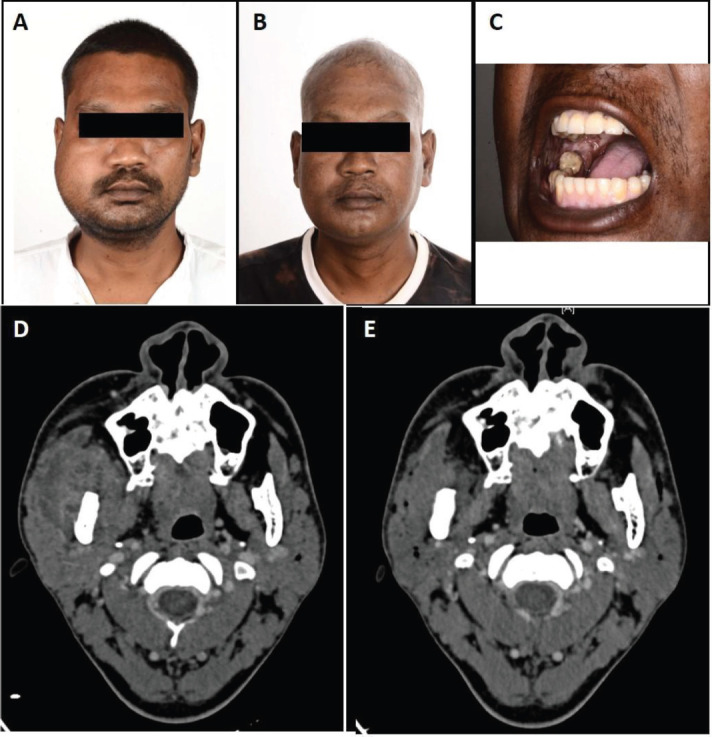
Clinico-radiological response after NACT. (a): Pre-chemotherapy image showing oedema above zygoma and fullness in infratemporal fossa. (b and c): Post-chemotherapy image showing near complete resolution of peri-tumoral oedema and buccal mucosa lesion, respectively. (d): Pre-chemotherapy CT image showing high ITF involvement. (e): Post-chemotherapy CT image showing significant reduction in tumour bulk and clearance of disease from high ITF.

After NACT, subjective benefit ≥ 50% was documented in 11 patients (79%; 95% CI = 57%–100%). Tumour of nine patients (65%; 95% CI = 39%–89%) showed partial response. Figure 1 shows the waterfall plot depicting depth of response after NACT. The median reduction in the size of measurable disease as per RECIST 1.1 was 42% (Range +7% to −70%). None of the patients had tumour progression. Figure 2 shows clinical and radiological response after NACT in one of the patients.

### Resection rate

Nine patients (65%; 95% CI = 39%–89%) were deemed resectable after NACT. Among these nine patients, eight underwent surgery, while one defaulted treatment. Tumour of one patient showed a pathological complete response. Six patients received adjuvant chemoradiation, while two patients defaulted adjuvant treatment. Of five patients who had unresectable tumour after NACT, two received radical chemoradiation, while the remaining three received palliative chemotherapy with paclitaxel and carboplatin.

### Survival

**Figure 3.**
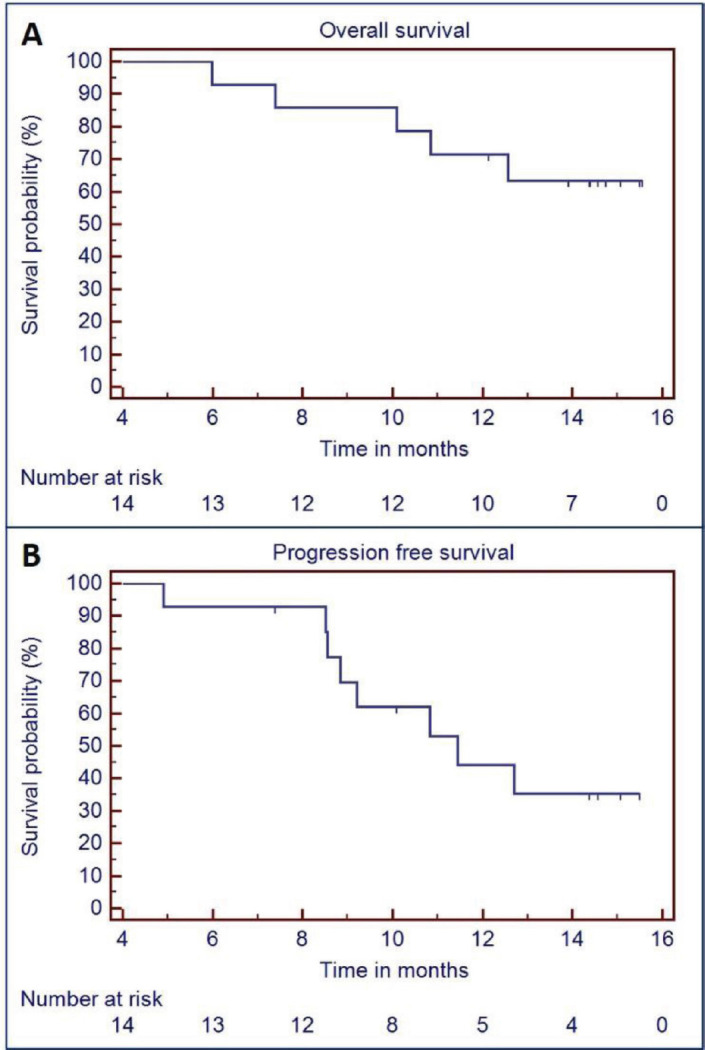
OS (a) and PFS (b) curves.

Median follow up was 14.6 months (95% CI = 14.1–15 months). Overall, eight patients had local progression of tumour. Of all the patients who underwent surgery (*n* = 8), two patients had local progression of tumour. One of these two patients had defaulted adjuvant chemoradiation. Median PFS was 11.4 months (95% CI = 7.9–15 months). There were four deaths, two deaths were due to aspiration pneumonia and other two deaths were due to disease progression. Median OS was not reached and OS at 15 months was 63.5% (95% CI = 37.8%–89.2%). Figure 3 depicts PFS and OS curves. Survival and response achieved with this regimen is summarized in Table 2.

### Compliance and adverse events

The median number of NACT administered was 3 (range = 2 to 4). There was a delay of ≥ 7 day in starting chemotherapy for three patients due to toxicities in two patients (grade 3 febrile neutropenia and candidemia) and due to logistics in one patient. Dose reduction to 75% of the planned dose was done in one patient due to toxicity (candidemia/grade 4 thrombocytopenia).

Adverse events during chemotherapy are summarized in Table 3. Grade 3/4 haematological toxicity was observed in eight patients (57%). Febrile neutropenia occurred in two patients (14%) which required in-patient supportive care and one of them developed candidemia, both patients had an uncomplicated recovery. Grade 3/4 non-haematological toxicity was observed in five patients (36%). Common grade 3 non-hematologic toxicities were diarrhoea (14%), mucositis (7%) and hypokalaemia (14%). Four patients developed grade 1 skin rash during NACT.

## Discussion

Triple metronomic chemotherapy leads to an exciting response rate (overall response rate = 43%) in platinum-refractory head and neck cancer, and thus it was considered as an appropriate adjunct to paclitaxel carboplatin for NACT regimen [10]. The mechanism of action of metronomic and MTD regimens are different and their side effects are non-overlapping, and hence we tested this regimen in fourteen patients in whom TPF/TP regimen could not be administered. The response rate achieved with this regimen was similar to that observed with TPF in phase 3 trials [13, 14]. In our study, only two out of eight patients (25%) who underwent surgery had local progression of tumour, while the rest of the six patients who received non-surgical treatment (chemoradiation or palliative chemotherapy/radiotherapy) experienced tumour progression. Retrospective studies comparing surgery versus definitive chemoradiation in OSCC have shown better disease control and survival with surgery [15, 16]. Iyer *et al* [17] in the subset analysis of their randomized trial reported favourable survival with surgery followed by radiotherapy when compared to chemo-radiation for tumours of oral cavity and maxillary sinus sites. This underscores the importance of surgery in multimodal management of locally advanced OSCC. Our study regimen led to a resectability rate of 65% with tumours of nine patients becoming resectable after NACT.

Median PFS and OS at 15 months achieved in our study is similar to that reported by Vermorken *et al* [13] in a phase 3 trial of TPF regimen. Haematological adverse events observed in our study were lower than that reported in phase 3 trials of TPF [13, 14]. The results suggested that the regimen is tolerable and causes modest immunosuppression with uncomplicated recovery in the patients. This regimen was found to have good response and resectability rate with acceptable toxicity. Thus, suggesting that this regimen could be tested further in head and neck cancer and can be used in COVID-19 pandemic times.

TPF chemotherapy is considered as the standard regimen for NACT in unresectable head and neck cancer and leads to higher response rate, OS than PF regimen [13, 14]. Noronha *et al* [18] have shown higher response rate with TPF chemotherapy when compared to the taxane-platinum containing regimen in technically unresectable borderline cancers. It was also noted that in two drug taxane-platinum regimens, docetaxel containing regimen (TP) has a higher response rate than paclitaxel containing regimen [18]. Hence, TPF or TP is the preferred regimen depending on financial affordability. TPF regimen leads to a high rate of adverse events and has a finite risk of treatment-related mortality [13, 14]. Indian patients in particular experience higher grade or 4 toxicities with TPF (~80%) and may require hospitalization and monitoring beyond completion of chemotherapy [19]. Caudell *et al* [20] showed that induction TPF chemotherapy in locally advanced head and neck cancer patients with low socio-economic status and poor performance status is associated with 15.3% mortality. Besides, as high as 38.2% patients could not complete planned treatment after induction TPF [20]. Thus, the administration of TPF in India is resource consuming, and hence is logistically not feasible in most of our patients due to low socioeconomic status. Administration of TPF/TP in such patients without adequate resources can lead to a higher rate of morbidity and mortality. Besides, selection of patients based on age, comorbidities, performance status and nutritional status is key for safe administration of TPF/TP. In the absence of financial aid, alternative regimens like paclitaxel-platinum have been used. Although this regimen has a lower rate of adverse events, it results in a lower rate of response and inferior outcomes. Hence, selection of an appropriate regimen in patients who are not fit for TPF/TP because of comorbidities or logistics is an issue.

This regimen, although not tested during the CORONA virus disease 2019 (COVID-19) pandemic, seems an attractive option during the pandemic. Due to the widespread pandemic, resources are allocated for the management of COVID-19 patients such as intensive care unit beds, ventilators, medical and paramedical staff. Administration of immunosuppressive TPF/TP regimen puts the patient at high risk of COVID-19 infection and higher case fatality due to COVID-19. Hence, the use of alternative neoadjuvant regimens such as the current regimen can be explored in this period.

The study has its own limitations. It is a small retrospective study and needs further testing before preferring it over TPF in normal circumstances. The study was performed in a uniform cohort of T4 oral cancers which are locally advanced and hence the results are applicable mainly in the oral cancer cohort. The sample size was small and the follow up duration was short. Besides, oral formulations were used in the study which limits its use in patients with nasogastric tube feeding.

## Conclusions

The combination of triple metronomic with paclitaxel-carboplatin leads to favourable response rate and survival with acceptable adverse events profile. This regimen underscores the synergy of metronomic and MTD scheduling in overcoming chemotherapy resistance. It can be tested further in randomized setting. However, in the current COVID-19 pandemic, this can be an alternative for patients in whom administration of TPF regimen is a concern.

## Conflicts of interest

None

## Funding declaration

No funding to declare.

## Figures and Tables

**Supplementary Table 1.** Details of clinico-radiological factors precluding upfront resection.

Case	Reason for technical unresectability
Patient 1	High ITF involvement
Patient 2	Peritumoral oedema up to zygoma
Patient 3	High ITF involvement
Patient 4	Peritumoral oedema up to zygoma
Patient 5	Peritumoral oedema up to zygoma
Patient 6	Peritumoral oedema up to zygoma
Patient 7	Posterior extent up to valleculla
Patient 8	Nodal disease up to 180° encasement of ICA
Patient 9	Nodal disease up to 180° encasement of ICA
Patient 10	High ITF involvement
Patient 11	Peritumoral oedema up to zygoma
Patient 12	High ITF involvement
Patient 13	High ITF involvement
Patient 14	Posterior extent up to valleculla

**Table 1.** Baseline characteristics.

Characteristic	Value
Age	38 years (29 to 59 years)
Sex Male Female	12 (86%) 2 (14%)
Comorbidities Diabetes mellitus Hypertension	2 (14%) 1 (1%)
ECOG performance status 0 1	6 (43%) 8 (57%)
Tumour site Buccal mucosa Oral tongue	12 (86%) 2 (14%)
T stage^a^ T4a T4b	4 (29%) 10 (71%)
N stage^a^ N0 N1 N2 N3	2 (14%) 4 (29%) 4 (29%) 4 (29%)
Stage group^a^ IVA IVB	2 (14%) 12 (86%)
Histology^b^ WDSCC MDSCC PDSCC SCC	3 (21%) 6 (43%) 2 (14%) 3 (21%)

a AJCC 8th edition

b Histology

WDSCC, Well differentiated squamous cell carcinoma; MDSCC, Moderately differentiated squamous cell carcinoma; PDSCC, Poorly differentiated squamous cell carcinoma; SCC, Squamous cell carcinoma

**Table 2.** Survival and response.

Variable	Value
**OS**	
Median OS – month	Not reached
15-month OS	63.5% (95% CI = 37.8%–89.2%)
**PFS**	
Median PFS – month	11.4 (95% CI = 7.9–15)
15-month PFS – %	35.4 (95% CI = 8.1%–62.6%)
**Response rate**	
Partial response – %	65% (9 patients)
Stable disease – %	35% (5 patients)
**Resectability – %**	65% (9 patients)

**Table 3.** Adverse events as per CTCAE 5.0.

Adverse events	Grade 2	Grade 3	Grade 4
Haematological Anaemia Neutropenia Thrombocytopenia Febrile neutropenia	5 (36%) 3 (21%) 1 (7%) -	1 (7%) 4 (29%) 2 (14%) 2 (14%)	0 (0%) 4 (29%) 1 (7%) 0 (0%)
Non haematological CINV^a^ Diarrhoea Mucositis Peripheral sensory neuropathy Hyponatremia Hypokalaemia Hypomagnesemia AST^b^ derangement ALT^c^ derangement	1 (7%) 2 (14%) 4 (29%) 1 (7%) 3 (21%) 0 (0%) 0 (0%) 0 (0%) 0 (0%)	0 (0%) 2 (14%) 1 (7%) 0 (0%) 0 (0%) 2 (14%) 1 (7%) 1 (7%) 1 (7%)	0 (0%) 0 (0%) 0 (0%) 0 (0%) 0 (0%) 0 (0%) 0 (0%) 0 (0%) 0 (0%)

a CINV – Chemotherapy induced nausea and vomiting

b AST – Aspartate aminotransferase

c ALT – Alanine aminotransferase
